# Effects of branched-chain amino acids on the muscle–brain metabolic axis: enhancing energy metabolism and neurological functions, and endurance exercise in aging-related conditions

**DOI:** 10.3389/fnut.2025.1709867

**Published:** 2025-12-03

**Authors:** Jing Wu

**Affiliations:** Ministry of Physical Education, Zhengzhou Health College, Zhengzhou, Henan, China

**Keywords:** branched-chain amino acids, muscle–brain metabolic axis, energy metabolism, neurological functions, aging

## Abstract

Aging is related to a decline in muscle mass, cognitive capabilities, and exercise proficiency, all of which collectively diminish quality of life and heighten the susceptibility to chronic illnesses. Branched-chain amino acids (BCAAs), with specific emphasis on leucine, have surfaced as pivotal regulators of muscle metabolism and may significantly influence the muscle–brain metabolic axis, thereby affecting hippocampal plasticity and cognitive wellbeing. This review consolidates contemporary evidence from 21 clinical and experimental investigations examining the influences of BCAA intake in conjunction with exercise on muscle functionality, cognitive outcomes, and endurance capacity in conditions associated with aging. The results indicate that BCAA supplementation, particularly when augmented with leucine and combined with resistance or multicomponent exercise regimens, facilitates muscle protein synthesis, mitigates inflammation, and enhances physical performance in the elderly population. Initial data also suggest possible advantages for hippocampal plasticity and cognitive function; however, this domain necessitates further investigation. The presence of challenges such as variability in study methodologies, supplementation regimens, and demographic characteristics of participants constrains the ability to draw definitive conclusions. Subsequent research endeavors ought to concentrate on clarifying the mechanistic pathways that connect muscular and cognitive health, refining supplementation methodologies, and investigating tailored strategies to alleviate age-associated sarcopenia and cognitive deterioration. In summary, the combination of BCAAs with physical exercise presents a potentially effective intervention to facilitate healthy aging through the interconnected metabolic axis of muscle and brain.

## 1 Introduction

As the global population ages, maintaining physical and cognitive health has become a major public health priority. Aging is frequently accompanied by sarcopenia, a progressive decline in skeletal muscle mass and strength, and by cognitive impairment that may progress to neurodegenerative diseases such as Alzheimer’s and Parkinson’s. Once viewed as separate conditions, these phenomena are increasingly recognized as interconnected through the muscle, brain metabolic axis, a bidirectional communication network integrating metabolic, endocrine, and neural signaling between skeletal muscle and the central nervous system (1–4). Skeletal muscle functions not only as a mechanical organ but also as an endocrine organ, releasing myokines and metabolites that influence brain metabolism and plasticity, while the brain regulates muscle activity via neural and hormonal pathways. With advancing age, this cross-talk becomes impaired, leading to frailty, fatigue, insulin resistance, mitochondrial dysfunction, and neuroinflammation (3, 4).

Among strategies proposed to restore this balance, nutritional interventions, particularly with branched-chain amino acids (BCAAs) such as leucine, isoleucine, and valine, have attracted substantial attention. These amino acids are abundant in skeletal muscle and are directly metabolized there via branched-chain aminotransferase (BCAT) and branched-chain α-keto acid dehydrogenase (BCKDH). Leucine in particular activates the mTORC1 pathway, promoting protein synthesis and mitochondrial biogenesis (5, 6). Beyond muscle, BCAAs also influence brain metabolism, serving as precursors for glutamate and GABA, key neurotransmitters that regulate cognition and mood (7–9). Dysregulated BCAA metabolism during aging, especially due to reduced BCKDH activity, can lead to the accumulation of toxic intermediates that impair insulin signaling and mitochondrial function (10, 11).

Nevertheless, appropriate supplementation has shown promising results in both human and animal models, suggesting enhancements in mitochondrial biogenesis, reductions in inflammation, and potential support for endurance and cognitive performance (12, 13). Because both skeletal muscle and the brain rely heavily on mitochondrial energy production, restoring BCAA metabolism may benefit both systems simultaneously.

Exercise, another proven non-pharmacological approach, interacts synergistically with BCAAs. During endurance or resistance exercise, muscles oxidize BCAAs for energy, while training upregulates enzymes of BCAA catabolism (14, 15). Thus, combining exercise with BCAA intake may amplify metabolic and functional gains, especially in older adults with diminished exercise tolerance and anabolic sensitivity. In summary, aging disrupts the metabolic communication between muscle and brain, contributing to sarcopenia and cognitive decline. BCAAs, particularly leucine, may help restore this axis by enhancing mitochondrial efficiency, protein synthesis, and neurotrophic signaling. The following sections examine (1) the metabolic characteristics and mechanisms of BCAAs, (2) their regulatory roles within the muscle–brain axis, and (3) the combined effects of BCAA supplementation and exercise on energy metabolism, physical function, and cognitive health in aging populations.

## 2 Branched-chain amino acids: metabolic characteristics and mechanisms

Branched-chain amino acids (BCAAs) (i.e., leucine, isoleucine, and valine) are essential amino acids that play key roles in muscle metabolism and whole-body nitrogen balance. BCAAs are predominantly catabolized in skeletal muscle rather than the liver, where they serve as substrates for energy production and as signals that regulate protein turnover (16, 17). The initial step of BCAA catabolism is reversible transamination by branched-chain aminotransferase (BCAT), producing branched-chain α-keto acids that are then oxidized by the branched-chain α-keto acid dehydrogenase complex (BCKDH). BCKDH activity is the rate-limiting control point for BCAA oxidation and is tightly regulated by phosphorylation–dephosphorylation and interacting regulatory proteins (18, 19). Disruption of BCKDH regulation or other defects in BCAA catabolism have been associated with elevated circulating BCAAs and altered metabolic signaling in aging and metabolic disease (20). Leucine is the most potent BCAA for activating anabolic signaling through mTORC1, thereby stimulating muscle protein synthesis and reducing proteolysis. Exercise and training increase muscle capacity for BCAA oxidation and enhance the protein-synthetic response to leucine and protein feeding (21, 22). BCAA catabolism also supplies tricarboxylic-acid (TCA) cycle intermediates (for example, acetyl-CoA and succinyl-CoA), linking amino-acid turnover to ATP production during recovery and endurance activity (23). Experimental evidence indicates that targeted BCAA or leucine supplementation can support mitochondrial function and aspects of muscle bioenergetics in aging models, although effects depend on dose, duration, and context (24–26). These findings suggest that BCAAs may help ameliorate age-related mitochondrial dysfunction and energy deficits in both muscle and brain tissues, reinforcing their role as key modulators of the muscle–brain metabolic axis. Collectively, these mechanisms illustrate how BCAAs serve as essential metabolic intermediates that sustain muscle anabolism, mitochondrial health, and neurotransmitter balance, forming the biochemical foundation of the muscle–brain metabolic axis.

## 3 The muscle–brain axis: crosstalk and coordination

The muscle-brain axis signifies a sophisticated, bidirectional communication framework that interconnects skeletal muscle metabolism with cerebral function, yielding significant consequences for aging and metabolic wellbeing. The muscle–brain metabolic axis refers to the bidirectional communication network through which skeletal muscle and the central nervous system coordinate energy metabolism, signaling, and functional adaptation. This axis is mediated by metabolites (such as amino acids and lactate), endocrine factors including myokines (e.g., IL-6, irisin, and BDNF), and neural pathways that integrate peripheral and central energy demands. Within this framework, BCAAs act as metabolic and signaling intermediates capable of influencing both muscular and neuronal bioenergetics, thereby providing a biochemical basis for the “muscle–brain” linkage emphasized in this review.

This axis integrates neural, endocrine, and metabolic pathways through which skeletal muscles exert influence over brain physiology and vice versa. This communication relies on muscle-derived myokines that modulate brain metabolism and plasticity. BCAAs, due to their metabolic and signaling functions, arise as pivotal regulators of this axis, facilitating the intricate interactions that preserve energy homeostasis and neurological health (27, 28). Myokines, including interleukin-6 (IL-6), irisin, and brain-derived neurotrophic factor (BDNF), released in the context of muscle contraction, exert influence on CNS functioning by traversing the blood–brain barrier (BBB) or by acting upon peripheral nerves. BCAAs have been reported to modulate the synthesis and secretion of these myokines, thereby augmenting the muscle’s communicative ability with the CNS. For example, the administration of leucine supplements has been shown to elevate the expression of BDNF in both muscular and cerebral tissues, thereby facilitating synaptic plasticity and enhancing cognitive functions (29, 30). Furthermore, BCAAs may exert regulatory effects on neurotransmitter biosynthesis and neuromodulation by acting as precursors for glutamate and GABA within the brain, consequently affecting the excitatory-inhibitory equilibrium and the stability of neural networks (21). BDNF, an essential neurotrophin implicated in neuronal survival, differentiation, and synaptic plasticity, is profoundly affected by the levels of BCAAs and the activity of muscle tissues. Research indicates that BCAAs promote BDNF expression via mTOR-dependent signaling pathways, thereby establishing a link between the availability of peripheral amino acids and central neuroplasticity responses (31).

This neurotrophic support is particularly crucial during the aging process, as reductions in BDNF have been related to cognitive deficits and an elevated risk of neurodegenerative disorders. The BCAA-induced enhancement of BDNF may therefore signify a therapeutic avenue through which nutritional and physical exercise interventions can sustain cognitive capabilities (32). Beyond neurotrophic modulation, BCAAs also influence neurovascular coupling and cerebral bioenergetics, fundamental elements of brain health and functionality. Neurovascular coupling guarantees the accurate regulation of cerebral blood flow in alignment with neuronal activity, thereby ensuring the appropriate provision of nutrients and oxygen. The metabolism of BCAAs affects this mechanism by regulating endothelial function and mitochondrial energy generation in both muscular and neuronal cells (33). For instance, the supplementation of BCAAs has been shown to promote mitochondrial biogenesis and enhance functionality within neuronal tissues, thereby augmenting ATP availability and mitigating oxidative stress, which jointly support neuronal survival and operational integrity (25). BCAAs may also improve neurovascular function and help preserve cognitive resilience in aging (34). Collectively, these findings emphasize the essential contribution of BCAAs within the muscle–brain metabolic axis, promoting a communicative framework that undergirds energy metabolism, neuroplasticity, and vascular health. This intercommunication is particularly salient in the context of aging, wherein perturbations in amino acid metabolism, myokine signaling, and neurovascular coupling are implicated in frailty, cognitive deterioration, and diminished endurance capacity. Leveraging the modulatory potential of BCAAs through dietary and exercise interventions presents a promising avenue for alleviating these age-associated challenges by reinstating and enhancing the muscle–brain dialogue (35, 36). In summary, BCAAs contribute to muscle–brain communication by regulating myokine release, neurotrophic signaling, and cerebral energy balance, emphasizing their integrative role in maintaining metabolic and cognitive health during aging.

## 4 Effects of BCAAs on energy metabolism in aging

Aging is correlated with progressive reductions in metabolic adaptability, mitochondrial operational efficiency, and skeletal muscle mass, collectively contributing to sarcopenia and heightened susceptibility to metabolic disorders. A defining characteristic of aging is the disruption of energy homeostasis, wherein diminished mitochondrial biogenesis and impaired oxidative phosphorylation adversely affect ATP synthesis and muscular functionality. These deficiencies not only contribute to physical frailty and diminished endurance but also engender systemic repercussions, encompassing cognitive deterioration and insulin resistance. BCAAs have emerged as pivotal regulators of metabolic health throughout the aging process, with an accumulation of evidence substantiating their roles in the restoration of mitochondrial functionality, the enhancement of energy production, and the amelioration of glucose metabolism in aged muscular and cerebral tissues (5, 37). Sarcopenia stems largely from mitochondrial dysfunction and anabolic resistance, the reduced responsiveness of aged muscle to nutrition and exercise. BCAAs, particularly leucine, may partially mitigate this resistance through the activation of mTORC1 signaling pathways, thereby facilitating protein synthesis and promoting mitochondrial biogenesis. Furthermore, BCAAs act as substrates for the TCA cycle, thereby bolstering energy production in the context of metabolic stress. In aging skeletal muscle, BCAA administration has been associated with increased muscle mass and strength, reduced fatigue, and delayed onset of sarcopenia-related disabilities (38, 39). Among the most compelling benefits of BCAAs within the framework of aging is their ability to enhance both mitochondrial biogenesis and functionality. The catabolism of BCAAs yields essential intermediates, such as succinyl-CoA and acetyl-CoA, that serve to replenish the TCA cycle and facilitate ATP production through oxidative phosphorylation. Notably, leucine specifically activates PGC-1α, which functions as a principal regulator of mitochondrial biogenesis.

The aforementioned activation precipitates an augmentation in the gene expression of mitochondrial transcription factors and respiratory enzymes, thereby enhancing mitochondrial density and functionality within aged skeletal muscle and neuronal tissues (26, 40). In experimental rodent models, supplementation with BCAAs has been correlated with improved mitochondrial respiration, diminished oxidative stress, and enhanced endurance performance, thereby underpinning its potential applicability as a therapeutic intervention for age-associated metabolic deterioration. A further significant facet of the aging process is the onset of insulin resistance, particularly within skeletal muscle, which is responsible for the predominant share of postprandial glucose uptake. While persistent elevations in circulating BCAA concentrations have been linked to insulin resistance in various metabolic disorders, regulated supplementation in older adults has exhibited seemingly paradoxical advantages. Research indicates that BCAAs, when administered at physiologically relevant dosages and in conjunction with physical activity or protein consumption, can enhance insulin sensitivity through mechanisms that promote glucose uptake, augment glycogen synthesis, and facilitate the translocation of GLUT4 (41, 42). The stimulation of the mTOR by leucine also contributes to the preservation of insulin signaling in senescent muscle; however, the long-term implications of this activation are contingent upon contextual factors, dosage, and the influence of other metabolic stressors. Notably, the effectiveness of BCAAs in the context of aging is markedly amplified when integrated with exercise regimens and additional dietary modifications. Resistance and endurance training enhance BCAA-driven anabolic and mitochondrial adaptations. Moreover, the synergistic interplay between exercise-induced myokines (such as IL-6 and irisin) and BCAA supplementation has been demonstrated to potentiate mitochondrial biogenesis and anti-inflammatory responses, thus enhancing both metabolic health and physical performance among older adults (43, 44). Nutritional methodologies that integrate BCAAs with diets abundant in protein or essential amino acid formulations yield superior advantages compared to the exclusive use of isolated BCAAs, thereby endorsing a more comprehensive paradigm for the preservation of energy metabolism and functional capabilities in aging demographics. In conclusion, BCAAs manifest intricate influences on energy metabolism throughout the aging process, predominantly by promoting mitochondrial integrity, enhancing protein synthesis, and facilitating insulin efficacy. When incorporated into systematic exercise and nutritional regimens, BCAA supplementation appears to be a promising intervention to help alleviate sarcopenia, enhance metabolic adaptability, and support functional autonomy in older adults. Overall, BCAA supplementation helps counteract age-related metabolic decline by promoting mitochondrial function, enhancing insulin sensitivity, and improving energy efficiency in both muscle and brain tissues.

## 5 Effects of BCAAs combined with exercise on quality of life, physical function, and muscle health in older adults

**Figure 1:**
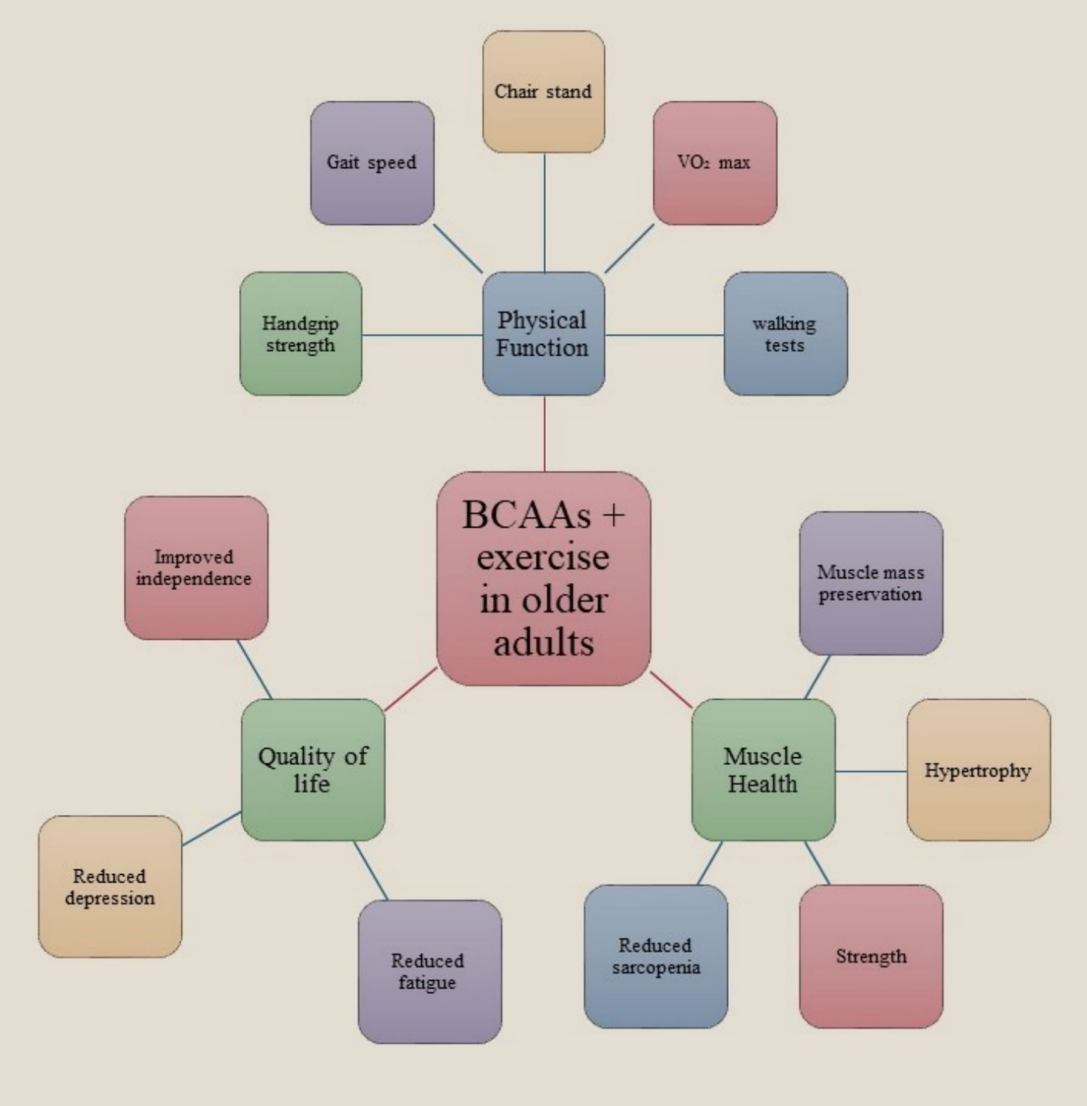
Effects of BCAA supplementation combined with exercise on physical function, muscle health, and quality of life in older adults. The integration of BCAAs with structured exercise enhances handgrip strength, gait speed, and endurance, preserves muscle mass, reduces fatigue and depressive symptoms, and supports independence, contributing to healthier aging.

The integration of BCAAs with physical exercise has demonstrated considerable advantages for the elderly population by augmenting physical functionality, enhancing quality of life (QoL), and fostering muscular health. Empirical investigations have indicated that senior individuals who engaged in BCAA supplementation in conjunction with regular physical activity exhibited significant advancements in strength, mobility, and endurance (45–47). This encompasses heightened handgrip strength, expedited chair stand performance, and enhanced ambulation speed. These physical enhancements contribute to improved overall functional capacity, facilitating the execution of daily activities and mitigating the likelihood of falls or injuries. Beyond the physical advantages, BCAA supplementation paired with exercise has been evidenced to alleviate fatigue and depressive manifestations in older adults, culminating in a substantial enhancement in overall QoL. The alleviation of fatigue enables older individuals to sustain elevated levels of activity and participation in social and recreational endeavors, which is pivotal for mental and emotional wellbeing. From a muscle health standpoint, BCAAs are fundamental in initiating muscle protein synthesis and facilitating energy production during physical exertion. This biochemical action can mitigate age-associated muscle degeneration (sarcopenia), conserve muscle mass, and enhance muscular strength. The synergistic effect of BCAA supplementation alongside resistance training is particularly advantageous for older populations, as it fosters muscle hypertrophy and preservation, which are essential for sustaining mobility and autonomy. In summary, the integration of BCAA supplementation with consistent physical activity presents a viable approach to promote healthier aging by augmenting physical performance, improving life quality, and ensuring muscle integrity. Nonetheless, it is imperative to utilize supplements under professional supervision and as an adjunct to a nutritionally balanced dietary regimen (Figure 1).

An investigation explored the impact of BCAAs in conjunction with physical exercise on fatigue, physical performance, and overall QoL among the elderly population. A cohort of 20 participants was allocated randomly to engage in an 8-week regimen of moderate-intensity aerobic and resistance training supplemented either with BCAAs (100 mg/kg/day) or a placebo. The evaluation of physical function was conducted through the assessment of handgrip strength, chair stand tests, gait velocity, VO_2_ max, and a 400-m walking test, while psychological outcomes were gauged through validated instruments measuring depression, fatigue, insomnia, and pain. The group receiving exercise in combination with BCAAs exhibited significantly enhanced outcomes in strength, mobility, and endurance in comparison to the exercise plus placebo group, in addition to substantial reductions in fatigue and symptoms of depression. Furthermore, noteworthy enhancements in the severity of insomnia and overall QoL were recorded in association with BCAA supplementation (45) (Table 1).

**Table 1:** Effects of BCAAs combined with exercise on physical function, quality of life, and muscle health in older adults.

Participants	Intervention	Physical function measures	Key physical outcomes	Other outcomes	Ref
20 older adults (63% female; 70.5 ± 1.2 yrs; BMI 35 ± 2 kg/m^2^)	Exercise + BCAAs (100 mg/kg/day) vs. Exercise + Placebo, 8 weeks	Handgrip strength, chair stands, gait speed, VO_2_ max, 400 m walk	Significant gains in handgrip strength, chair stands, and 400 m walk time	ISI ↓ 30%, fatigue ↓ 21%, QoL ↑ 16% with BCAAs; fatigue ↓ 45% with BCAA vs. +92% ↑ placebo; depressive symptoms ↓ 29% with BCAA vs. +5% ↑ placebo	(45)
66 community-dwelling adults ≥50 yrs (68.1 ± 7.1 yrs; 16.7% male)	Sarcojoint® (1 pack twice daily) + resistance exercise (45 min gym + 2 × 30 min home/week) vs. placebo + same exercise	30-s chair-rise, gait speed, handgrip strength	Increased mid-thigh muscle mass; no significant difference in strength or performance	↓ body fat %, ↑ serum vitamin D; MRI subgroup: improved muscle mass, ↓ intramuscular fat	(13)
35 frail older adults (83 ± 3 yrs, residential care homes)	Four groups: (i) ME + BCAA, (ii) ME, (iii) BCAA, (iv) Control; 40-week protocol (16 wk intervention + 8 wk washout + 16 wk retraining)	SPPB, Fried’s frailty phenotype	Exercise improved function and prevented frailty progression; BCAA alone had no effect but reduced frailty short-term and mitigated detraining loss	Salivary testosterone correlated with grip strength; no changes in mood or cognition	(58)
100 community-dwelling adults (68.7 ± 5.8 yrs; 52% female; BMI 27.1 ± 5.2 kg/m^2^)	Four groups: Control, Exercise (E), Exercise + Protein (EP), Protein only (P); 16 weeks; E/EP = resistance 2 × + circuit 1 × per week; EP/P = leucine-enriched whey (1.5 g/kg/day)	Cardiometabolic health markers	↓ LDL-C and insulin in EP and P vs. control; improved HOMA-IR in both	↓ resistin in the P group; kidney function unchanged	(59)
35 frail older adults (≥75 yrs; ME + BCAA = 8, ME = 7, BCAA = 7, Control = 13)	40-week multicomponent exercise (ME) ± BCAA; includes washout phase	Lower-limb strength, cognitive profile, and frailty tests	ME + BCAA improved cognition, ↑ albumin linked to muscle strength, ↓ TNF-α	Minor improvements in inflammation and function; better independence and QoL	(60)
28 post-hospitalized older adults	Resistance training 2×/week + leucine-enriched whey vs. placebo, 12 weeks	Upper/lower body strength, aerobic capacity, SPPB	Improved function in both groups; no extra effect of protein; no muscle mass gain	Nutritional status and body composition are unchanged	(61)
Frail/pre-frail older adults in long-term care	Crossover: exercise 2×/week + BCAA (6 g pre-exercise) vs. exercise + placebo; 3-month cycles with washout	Isometric limb strength, Functional Reach Test, Timed Up & Go, activity level	~10% ↑ in lower-limb strength and FRT during BCAA phases only	Improved dynamic balance and strength; intervention feasible and effective	(46)
30 older adults (leucine *n* = 15, control *n* = 15)	Leucine 10 g/day vs. placebo + resistance training, 12 weeks	Leg strength, chair stands, TUG test, functional status	Both groups improved; the leucine group showed greater gains in chair stands and TUG	Body composition, QoL, depression, and diet are unchanged	(47)
35 frail adults (83–93 yrs; MEP + BCAA = 8, MEP = 7, BCAA = 7, Control = 13)	40-week elastic-band multicomponent program ± BCAA (intervention-washout-intervention design)	Fried’s frailty phenotype, nutritional, and anthropometric data	Frailty stable in MEP ± BCAA; worsened in controls; MCH/MCHC maintained	Suggests pro-immune effects and supports nutrition + exercise for healthy aging	(62)
81 adults ≥65 yrs with sarcopenia (RT + PRO = 27, RT = 27, PRO = 27)	24-week resistance training ± leucine-enriched whey (11 g protein + 2.3 g leucine) 2×/week; + 24-week de-training	ASMI, handgrip strength	RT + PRO ↑ ASMI and HGS at 24 wk; maintained higher values post de-training vs. RT alone	The combined intervention provided better long-term maintenance	(63)
178 pre-frail adults (112 C, 44 Nu, 22 Nu + Ex)	3-month leucine-enriched protein ± exercise	SPPB, gait speed, grip strength, 5 × sit-to-stand	Nu + Ex ↑ gait speed, 5 × STS, SPPB, FFM, ASM; Nu and Nu + Ex ↓ IL-6, TNF-α	Anti-inflammatory effects not sustained at 6 mo; Nu + Ex improved mood and self-perceived health	(64)
18- and 2-month-old C57BL/6 mice (YC = 8, AC = 8, AMI = 8)	AMI: 5% leucine diet + combined aerobic (swimming 30 min) + resistance (jumping 40–50% BW); AC/YC: alanine diet (3.4%), no exercise	Grip strength, gastrocnemius weight, muscle/body-weight ratio	AMI ↑ muscle weight, protein synthesis, phosphorylation of mTOR, p70S6K, 4E-BP1, PI3K III; ↓ Sestrin2; ↑ grip strength	AMI ↑ plasma leucine & ↓ cortisol; supports BCAA + exercise for muscle health during aging	(65)

Although Sarcojoint® is a composite supplement, its core formulation is based on BCAAs and related anabolic cofactors; therefore, it provides an example of how BCAA-enriched interventions, when combined with resistance exercise, may influence muscle health in older adults. A trial using Sarcojoint®, a BCAA-based supplement, combined with resistance training in adults over 50 years, found modest gains in muscle area and vitamin D levels but no significant changes in strength (13). These findings, while derived from a mixed formulation, further support the notion that BCAA-enriched supplementation synergizes with exercise to promote muscle preservation in aging populations.

Similar RCTs testing leucine-enriched or multicomponent interventions (45, 48) consistently show that BCAA intake alongside exercise improves strength, mobility, and fatigue in older adults. Overall, the integrated application of BCAAs supplementation alongside physical exercise reveals significant advantages in enhancing physical function, promoting muscular health, and improving the overall QoL among elderly individuals. Empirical evidence indicates that BCAAs, with a particular emphasis on leucine, facilitate muscle protein synthesis and amplify the anabolic response to both resistance and aerobic activities. This synergistic methodology may serve to mitigate age-associated muscle degeneration (sarcopenia), augment strength, and foster functional autonomy. Nonetheless, there exists a necessity for further comprehensive, longitudinal investigations to refine dosing regimens and exercise methodologies to optimize clinical outcomes within this demographic. Taken together, the evidence supports that combining BCAAs with structured exercise produces synergistic effects on muscle strength, endurance, and quality of life among older adults. In summary, across diverse study designs, the concurrent use of BCAA supplementation and structured exercise consistently enhanced physical performance, muscle strength, and functional independence among older adults. While some interventions used multi-nutrient formulations, the collective evidence supports the central role of BCAAs, particularly leucine, in amplifying the anabolic and restorative effects of exercise on the muscle–brain axis.

## 6 Molecular and biochemical mechanisms of BCAAs in muscle and brain

**Figure 2:**
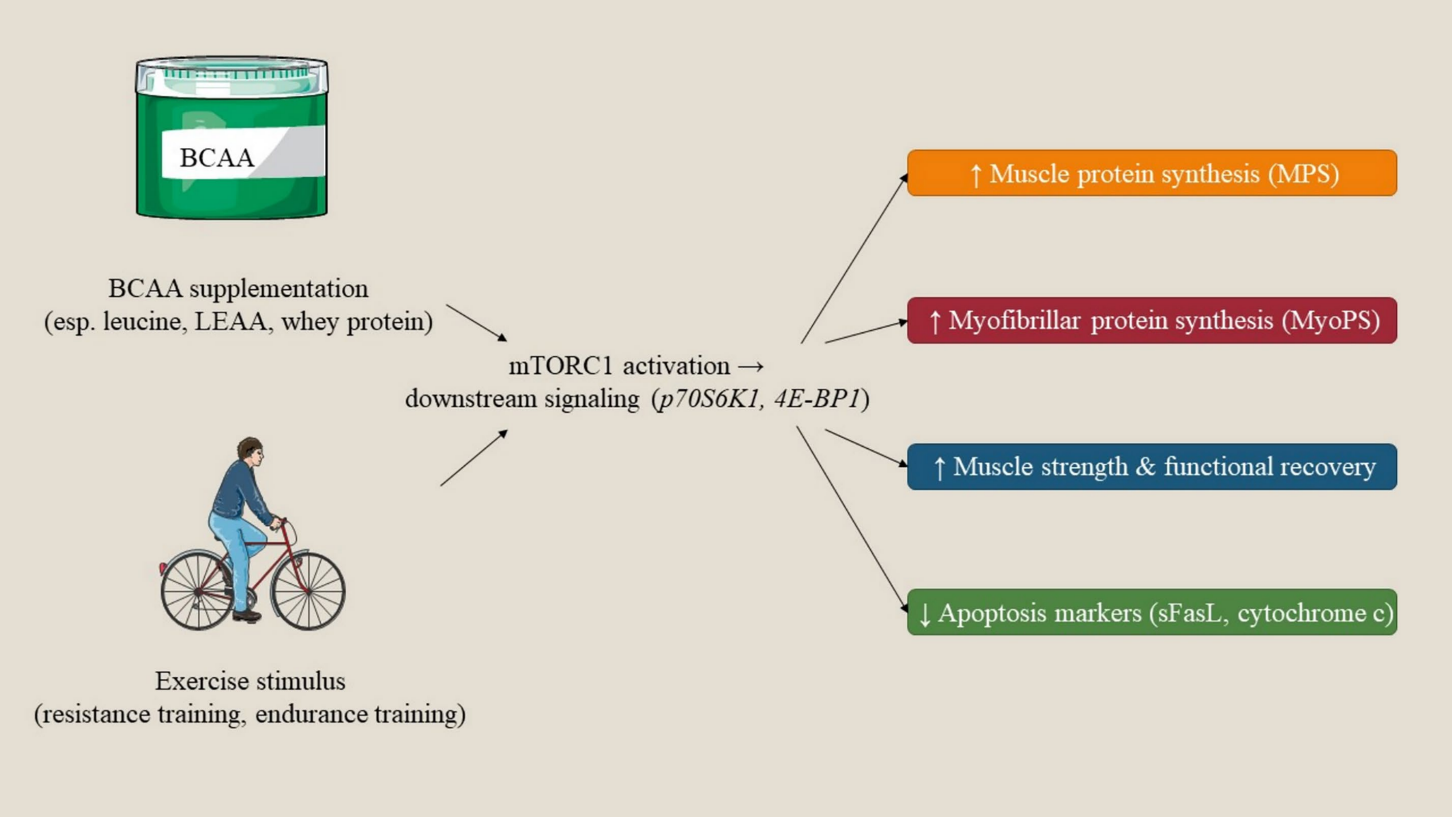
Proposed mechanistic pathways by which BCAAs and exercise promote anabolic responses in older adults. BCAA supplementation (particularly leucine) and exercise synergistically activate the mTORC1 signaling pathway (p70S6K1, 4E-BP1), leading to increased muscle protein synthesis, improved strength and recovery, and attenuation of apoptosis-related markers.

BCAAs play pivotal roles in the functioning of both muscular and cerebral systems through distinct molecular and biochemical mechanisms. In skeletal muscle, BCAAs act as fundamental regulators of protein biosynthesis and metabolic pathways associated with energy. Notably, leucine specifically activates the mTOR signaling cascade, thereby facilitating muscle protein synthesis and contributing to muscle repair and hypertrophy. Moreover, BCAAs function as critical substrates for ATP production during physical exertion, assisting in the alleviation of muscular fatigue and enhancing endurance capabilities. In the brain, BCAAs regulate neurotransmitter synthesis and cognitive activity. They engage in competitive interactions with aromatic amino acids at the BBB, thus modulating concentrations of neurotransmitters like dopamine and serotonin, which are instrumental in regulating mood, cognitive performance, and mental fatigue. In addition, BCAAs may help as nitrogen donors in the synthesis of glutamine and glutamate, both of which are vital for effective neurotransmission and cerebral metabolism. A comprehensive understanding of these molecular and biochemical pathways underscores the therapeutic implications of BCAAs in the context of muscle recovery, neuroprotection, and cognitive wellbeing (Figure 2).

Older adults generally demonstrate a diminished muscle protein synthesis (MPS) response to minimal protein intakes subsequent to exercise when juxtaposed with their younger counterparts. A research investigation was conducted to ascertain whether the co-consumption of 1.5 g of free leucine alongside 15 g of milk protein could augment post-exercise MPS in geriatric men. A cohort of 24 healthy older men (mean age 67) was randomly allocated to receive either 15 g of milk protein in isolation (15G) or in combination with supplementary leucine (15G + LEU) post resistance training. The researchers assessed protein catabolism, amino acid assimilation, overall protein metabolism, and MPS utilizing stable isotope tracer methodologies. The findings revealed that the incorporation of leucine significantly elevated post-exercise MPS rates by 16–19% in comparison to protein administration alone. These results indicated that leucine supplementation possesses the potential to enhance the anabolic response to protein consumption in older adults subsequent to resistance training (49) (Table 2). The implications of omega-3 fatty acids and BCAAs on apoptosis induced by resistance exercise in older adults remain ambiguous. In an investigation, 11 elderly men (mean age 62.8) engaged in two sessions of resistance exercise, each preceded by a one-week supplementation phase consisting of either 20 g of BCAA or 2,700 mg/day of omega-3 fatty acids. A three-week washout interval was instituted between the experimental trials. Apoptosis-related biomarkers, including soluble Fas ligand (sFasL), cytochrome c, Bax, Bcl-2, and NF-κB, were quantified prior to and following exercise. Resistance exercise elevated the levels of cytochrome c, sFasL, and Bax. Nonetheless, the significant elevation in sFasL was exclusively observed in the control trial and not in the supplementation trials. No noteworthy effects were identified concerning Bcl-2 or NF-κB. The results indicate that short-term supplementation with omega-3 or BCAA may partially mitigate the apoptosis markers elicited by resistance exercise in older men (50).

**Table 2:** Summary of interventions and outcomes on muscle protein synthesis and anabolic responses in older adults following BCAAs and exercise interventions.

Participants	Intervention	Key outcomes	Other outcomes	Ref
24 healthy older men (67 ± 1 yrs) randomized to 15 g milk-protein concentrate (15G; *n* = 12) or 15 g milk-protein + 1.5 g free leucine (15G + LEU; *n* = 12) after resistance exercise	Co-ingestion of 1.5 g free leucine with 15 g milk protein immediately post-exercise vs. protein alone	Myofibrillar protein-synthesis (MPS) ↑ 16% (L-[ring-^2^H₅]phenylalanine) and ↑ 19% (L-[1-^13^C]leucine) in 15G + LEU vs. 15G	Higher proportion of protein-derived AAs in plasma of 15G group, suggesting slightly reduced AA appearance with leucine co-ingestion, though digestion/absorption unaffected	(49)
11 older men (62.8 ± 2.2 yrs) in a randomized crossover trial	One-week supplementation with BCAA 20 g/day or *ω*-3 2.7 g/day → acute resistance exercise; 3-wk washout	Exercise ↑ apoptotic markers (sFasL, cytochrome c, Bax); sFasL rises significantly only in control, indicating both supplements attenuated apoptosis	No differences in NF-κB or Bcl-2; selective modulation of apoptotic signaling	(50)
24 older women (65 ± 1 yrs) randomized to LEAA₁.₅ (1.5 g), LEAA₆ (6 g), or whey protein (WP, 40 g); *n* = 8/group	Supplementation with LEAA₁.₅, LEAA₆, or WP ± leg extensions (6 × 8 reps @ 75% 1-RM)	Feeding increased MPS in all groups (0–2 h); LEAA₆ & WP maintained MPS 0–4 h; exercise further ↑ MPS in all	WP uniquely ↑ p-p70 S6K1 at 2 h (FED) and 2–4 h (FED-EX); plasma insulin & EAA levels higher/prolonged with WP; no effect on femoral/MBF	(51)
18 men (young 24 ± 6 yrs; older 70 ± 5 yrs; *n* = 9/group)	Post-exercise drink: 10 g protein + 24 g CHO + 4.2 g leucine (LEU) vs. isonitrogenous control + 4.2 g alanine (ALA)	In both age groups, MPS was higher after LEU vs. ALA (AUC 0–4 h, *p* < 0.05) and peak FSR ↑ (young 0.11 vs. 0.08%·h^−1^)	p70 S6K1 phosphorylation ↑ after both, but greater with LEU (3.2 vs. 2.2-fold, *p* < 0.05), especially in older men	(52)
16 older women (66 ± 2.5 yrs; *n* = 8/group)	20 g whey protein (WP) or 3 g LEAA (40% leucine) ± resistance exercise (6 × 8 @ 75% 1-RM)	Both WP & LEAA ↑ MPS 0–2 h; MPS remained elevated 0–4 h only after exercise; p70 S6K1 ↑ 2 h post exercise (*p* < 0.05); APS ↑ equally	Plasma insulin & EAA were higher after WP vs. LEAA; no changes in leg or microvascular blood flow except exercise-induced MBF ↑; no extra benefit of WP over LEAA	(53)
15 older men randomized: control (*n* = 7, EAAs 10 g + 1.85 g leu) vs. LEU group (*n* = 8, EAAs 10 g + 3.5 g leu)	EAAs with standard vs. leucine-enriched content taken 1 h post-exercise; biopsies at 0, 2, 5, 24 h	p70 S6K1 ↑ 2 h in both; 4E-BP1 ↑ only in LEU group; MyoPS ↑ ≈ 90% both at 5 h, but remained ↑ at 24 h only with LEU	AAT mRNA ↑ 2 & 5 h in both; sustained ↑ 24 h only with LEU; leucine prolonged anabolic signaling post-exercise	(54)
17 elderly men (63 ± 5 yrs; BMI 25 ± 2 kg/m^2^) randomized BCAA (*n* = 9) or placebo (*n* = 8)	Six-week endurance training (1 h/day, 4 d/week @ 75 ± 9% HRmax) ± oral BCAA (16 g leucine, 2 g isoleucine, 2 g valine)	Both groups ↑ VO_2_ max ≈ 5%; capillary density ↑ in both (type I fibers ↑)	No change in fiber-type distribution or CSA; BCAA did not enhance morphologic adaptations beyond training alone	(55)

Another investigation assessed the muscle protein synthesis (MPS) responses in elderly women participants to leucine-enriched essential amino acids (LEAA) in comparison to a substantial dosage of whey protein (WP). Subjects were randomly allocated to receive either 6 g LEAA, 1.5 g LEAA, or 40 g WP. MPS was quantified both at rest and following feeding alone (FED) or feeding in conjunction with resistance exercise (FED-EX). While plasma insulin and amino acid concentrations were elevated and persisted for a longer duration with WP, LEAA (particularly at the 6 g dosage) elicited comparable peak responses. All interventions resulted in an increase in MPS, with LEAA_6 and WP demonstrating prolonged effects over a span of 4 h. Nevertheless, when combined with resistance training, all cohorts exhibited analogous enhancements in MPS. Importantly, only WP significantly elevated the anabolic signaling marker p-p70S6K1. Collectively, even a modest dosage of LEAA (1.5 g) effectively stimulated MPS, indicating that the leucine content, rather than the total protein quantity, is crucial for optimizing the anabolic response in older women (51). Optimizing the anabolic responses to nutritional intake and resistance training is paramount for the preservation of muscle integrity across different age demographics. A research investigation evaluated the hypothesis that the incorporation of leucine into a protein beverage would augment muscle protein synthesis (MPS) following resistance training in younger (24 ± 6 years) and older male subjects (70 ± 5 years). Subjects were administered either a protein beverage enriched with 4.2 g of leucine (SFO + LEU) or an isonitrogenous control containing alanine (SFO + ALA) subsequent to exercise. Muscle biopsies were utilized to evaluate MPS and anabolic signaling (phosphorylation of p70S6K1) over a duration of 4 h. The results indicated that both younger and older male subjects exhibited significantly elevated MPS responses when leucine supplementation was employed in comparison to alanine. The phosphorylation of p70S6K1 was observed to increase in both cohorts, yet the enhancement was notably more significant in the leucine group, particularly among older male participants. These outcomes indicated that the enrichment of a submaximal protein dosage with leucine substantially bolsters the anabolic response to resistance training in both younger and older male subjects (52).

The anabolic responses to nutritional intake and physical exercise among older women remain inadequately elucidated; however, these responses are crucial for the mitigation of sarcopenia. A research investigation assessed the differential impacts of two distinct nutritional protocols, specifically, a bolus administration of 20 g of whey protein (WP) contrasted with a low-dose leucine-enriched essential amino acid supplement (LEAA; comprising 3 g with 40% leucine), on albumin protein synthesis (APS) and muscle protein synthesis (MPS) in a cohort of older women with an average age of 66 years. Evaluations were conducted at baseline, subsequent to feeding (FED), and following feeding combined with resistance exercise (EX). The administration of WP resulted in elevated plasma insulin and amino acid concentrations when compared to LEAA. Neither nutritional strategy influenced whole-leg blood flow; however, muscle microvascular blood flow exhibited a comparable increase following FED-EX in both treatment groups. Both WP and LEAA were found to equally stimulate MPS during the initial 2 h post-feeding, accompanied by a sustained elevation of MPS subsequent to FED-EX. Anabolic signaling through the phosphorylation of p70 S6K1 was observed to increase exclusively after FED-EX. APS demonstrated a comparable increase with both nutritional supplements (53). The post-exercise consumption of protein or amino acids is integral to the restoration of muscle protein synthesis (MPS) in the elderly population; however, the precise nutritional components implicated in this process remain inadequately elucidated. A separate investigation examined the influence of augmented leucine consumption on myofibrillar protein synthesis (MyoPS), anabolic signaling pathways, and the expression of amino acid transporters over a 24-h period following resistance exercise (RE) in older men. Subjects ingested 10 g of essential amino acids (EAAs) either with a standard leucine concentration (1.85 g) or a leucine-enhanced formulation (3.5 g) 1 h subsequent to RE. Muscle biopsy analyses indicated that both cohorts exhibited an increase in p70 S6 kinase 1 phosphorylation at the 2-h mark; however, phosphorylation of 4E binding protein 1 was observed to rise solely in the leucine-enriched cohort. MyoPS experienced an approximate elevation of 90% at 5 h for both groups, yet it remained significantly elevated at the 24-h mark exclusively in the leucine-enriched group. In a similar vein, the mRNA expression levels of amino acid transporters demonstrated early increases in both groups (54).

An investigation assessed the impact of a 6-week endurance training regimen supplemented with BCAAs on the structural characteristics of skeletal muscle in older men (mean age 63). The participants were stratified into two distinct cohorts: one cohort received a daily dosage of BCAA supplements (2 g isoleucine, 16 g leucine, 2 g valine), while the other cohort was administered a placebo. Both cohorts engaged in cycle ergometer training for 1 h per day, 4 days per week, at approximately 75% of their maximal heart rate. Muscle biopsies collected prior to and subsequent to the training regimen were scrutinized for fiber cross-sectional area, fiber type distribution, and capillary density. The endurance training intervention resulted in an approximate 5% enhancement in maximal oxygen uptake and significantly augmented muscle capillary density (number of capillaries per fiber and interaction with type I fibers) across both cohorts. Nevertheless, no alterations were observed in muscle fiber type distribution or fiber dimensions, and BCAA supplementation did not facilitate any of these training-induced histological adaptations (55). Overall, the molecular and biochemical pathways associated with BCAAs are essential for preserving muscular integrity and facilitating optimal cerebral function. Through the modulation of protein synthesis, energy metabolism, and the equilibrium of neurotransmitters, BCAAs play a pivotal role in promoting muscle hypertrophy, enhancing recovery, and supporting cognitive wellbeing. Ongoing investigations into these biological mechanisms may reveal novel therapeutic approaches for the treatment of muscular disorders and neurological ailments. In brief, these molecular pathways demonstrate how BCAAs modulate protein synthesis, neurotransmission, and mitochondrial activity, linking cellular metabolism to functional improvements in muscle and brain performance.

## 7 Combined supplementation: BCAAs with other nutrients

The integration of BCAAs with other essential nutrients, including omega-3 fatty acids, citrulline, and carnitine, has the potential to significantly augment their comprehensive benefits pertaining to both muscular and cognitive health. Omega-3 fatty acids facilitate anti-inflammatory mechanisms and enhance the functionality of cellular membranes, thereby aligning with the role of BCAAs in promoting muscle recovery as well as cognitive efficiency. Carnitine plays a pivotal role in the translocation of fatty acids into the mitochondria, thereby facilitating energy production and intensifying the effects of BCAAs on muscle metabolism and endurance. Together, these nutrients enhance muscle recovery and cognitive health.

An investigation was conducted to ascertain whether the combination of L-carnitine (LC) and leucine supplementation contributes to the enhancement of muscle strength and hypertrophy in older women participating in resistance exercise training (RET). A total of 37 healthy women, aged between 60 and 75 years, successfully completed a 24-week RET regimen, engaging in training sessions bi-weekly. The participants were classified into three distinct groups: one receiving LC + leucine (1 g LC + 3 g leucine per day), another administered leucine alone (4 g per day), and a control group that did not receive any supplementation. Muscle strength, assessed through isometric and isokinetic measurements, as well as thigh muscle volume, were evaluated pre- and post-intervention utilizing dynamometry and magnetic resonance imaging (MRI), respectively. All groups exhibited substantial improvements in both muscle strength and volume; however, the absence of significant intergroup disparities implies that supplementation did not augment the effects of RET. Plasma total carnitine concentrations demonstrated an increase solely in the LC + L group, alongside a notable elevation in trimethylamine N-oxide (TMAO) levels. Both groups receiving supplementation manifested an increase in serum decorin; however, no significant alterations were noted in insulin-like growth factor 1 (IGF-1) or myostatin levels (56) (Table 3). Another investigation assessed the implications of concurrent citrulline (CIT) and leucine (LEU) supplementation in conjunction with exercise on body composition and physical activity among elderly Japanese women with a low body mass index (16–21 kg/m^2^). Over a duration of 20 weeks, subjects ingested either CIT·LEU (0.8 g CIT + 1.6 g LEU, administered bi-daily; *n* = 10) or a placebo (*n* = 13), while engaging in a weekly exercise regimen lasting 75 min (which included weight-bearing and square stepping activities). Following the intervention, the CIT·LEU cohort exhibited statistically significant enhancements in body weight, BMI, body mass, household and overall physical activity, as well as plasma phenylalanine concentrations (*p* < 0.05), whereas the placebo group did not demonstrate any comparable advancements. These findings propose that the integration of CIT and LEU supplementation with infrequent exercise may facilitate improvements in body composition and physical activity levels in underweight elderly women, potentially aiding in the prevention of sarcopenia and frailty (57). A study examined the implications of omega-3 fatty acids in conjunction with BCAAs on apoptosis induced by resistance exercise in elderly men (mean age 62.8). Although exercise induced elevations in sFasL, cytochrome c, and Bax, this physiological response was diminished subsequent to supplementation with either omega-3 or BCAAs. No statistically significant alterations were observed in Bcl-2 or NF-κB across the experimental conditions (50). In totality, the combined supplementation of BCAAs with omega-3, citrulline, and carnitine presents synergistic advantages that enhance muscular performance, recovery processes, and cognitive health. This comprehensive approach promotes optimal metabolic and cognitive outcomes, rendering it a promising methodology for the enhancement of holistic physical and mental wellbeing. Collectively, the integration of BCAAs with nutrients such as omega-3 fatty acids, citrulline, and carnitine may amplify their anabolic, anti-inflammatory, and neuroprotective benefits.

**Table 3:** Combined supplementation: BCAAs with other nutrients.

Type of intervention	Dosage	Duration of study	Subjects	Results	Ref
Progressive resistance training (RET) + L-carnitine + leucine (LC + L group)	1 g L-carnitine-L-tartrate + 3 g L-leucine/day	24 weeks	12 healthy women, aged 60–75	RET increased muscle strength and volume; plasma carnitine and TMAO rose, but supplementation provided no additional muscle benefit.	(56)
Exercise + citrulline and leucine supplementation (Ex + CIT·LEU group)	~0.8 g citrulline + 1.6 g leucine, twice daily	20 weeks	10 older Japanese women with low BMI (16–21 kg/m^2^)	Significant gains in body weight, BMI, and physical activity; improved body composition and activity levels suggest potential protection against sarcopenia and frailty.	(57)
Acute resistance exercise + omega-3 supplementation	2,700 mg omega-3/day	1 week (crossover design)	11 older men (mean age 62.8 ± 2.2 years)	Omega-3 attenuated exercise-induced increases in apoptosis marker (sFasL); no effect on NF-κB or Bcl-2 expression.	(50)
Acute resistance exercise + BCAA supplementation	20 g BCAA/day	1 week (crossover design)	11 older men (mean age 62.8 ± 2.2 years)	BCAAs partially reduced exercise-induced apoptosis (sFasL); no significant effect on NF-κB or Bcl-2 expression.	(50)

## 8 Limitations, challenges, and key considerations in BCAA and exercise research

One of the predominant limitations and challenges in the scholarly investigation of BCAA supplementation in conjunction with exercise is the considerable variability inherent in study designs. While numerous studies adopt randomized controlled trials, which are regarded as the gold standard in clinical research, the restricted sample sizes and pilot-scale interventions diminish the statistical power and generalizability of the resultant findings. Design variability, such as crossover versus parallel trials, complicates comparison across studies. In addition, there exists a notable absence of standardized protocols for both exercise interventions and supplementation regimens, which hampers the ability to draw consistent conclusions across the existing body of literature. The duration of interventions is also characterized by significant variability, with timelines extending from a few weeks to several months. Short-term studies often fail to capture the full extent of physiological adaptations related to muscle and brain function, whereas longer-duration studies encounter issues such as participant adherence, dropout rates, and the necessity of controlling for confounding variables. Furthermore, a limited number of studies examine the ramifications of detraining or the persistence of benefits following the cessation of supplementation and physical activity, thereby leaving significant voids in the existing body of knowledge. The exercise modalities employed in these studies exhibit considerable variability, encompassing resistance training, ME interventions (which integrate aerobic, balance, and flexibility training), as well as endurance training. Such discrepancies impact the physiological responses elicited and the manner in which BCAAs influence muscle metabolism and cognitive functions. Generally, resistance training is recognized for its capacity to enhance muscle protein synthesis, whereas endurance training may facilitate mitochondrial adaptations and cognitive stamina; however, the interplay between the type of exercise and the effects of BCAAs remains inadequately investigated. The parameters surrounding the dosage, formulation, and timing of BCAA supplementation pose additional challenges. Research studies differ in the quantities of leucine-enriched BCAAs or whey protein administered, along with variations regarding the timing of supplementation—whether it is ingested prior to, during, or subsequent to physical exertion. Certain research protocols incorporate supplementary nutrients such as omega-3 fatty acids or L-Carnitine, which may obfuscate the interpretation of the findings. The inconsistent documentation of adherence and bioavailability further exacerbates the evaluation of the genuine effects of supplementation. Characteristics of participants, including age, sex, baseline fitness levels, health status, and frailty, exhibit considerable variability across studies. Although the majority of research is directed toward older adults, the criteria for inclusion vary widely, encompassing individuals from healthy to pre-frail or sarcopenic classifications, all of which may affect their responsiveness to interventions. Disparities in hormonal profiles, dietary consumption, and genetic predispositions also contribute significantly, rendering the generalization of results problematic and underscoring the necessity for personalized methodologies. The outcome measures employed differ markedly, with some investigations concentrating on physical performance indicators such as muscle strength or gait velocity, while others emphasize molecular biomarkers like muscle protein synthesis or inflammatory markers, and a limited number focus on cognitive or neuroplasticity outcomes. The lack of standardized biomarkers limits understanding of the muscle–brain mechanism. Furthermore, uncontrolled confounding variables such as dietary habits, pharmacological interventions, lifestyle choices, and placebo responses may skew results. Numerous studies fail to rigorously manage or disclose these variables, potentially compromising the integrity of the findings. It is imperative to ensure adequate blinding and control conditions to mitigate such biases. In conclusion, while extant evidence indicates possible advantages of BCAA supplementation in conjunction with exercise for enhancing muscle health and cognitive function in elderly populations, the domain is encumbered by methodological variability and practical obstacles. Addressing these limitations through expansive, longitudinal, and more standardized research with clearly defined populations and thorough outcome evaluations will be pivotal in enhancing both understanding and clinical implementation.

## 9 Conclusion and future directions

In conclusion, the existing corpus of literature indicates that BCAA supplementation in conjunction with physical exercise shows considerable potential to enhance strength, muscle mass, physical functionality, and possibly cognitive outcomes in older adults. Numerous investigations reveal that BCAAs, particularly those enriched with leucine, interact synergistically with resistance training or ME regimens to augment muscle protein synthesis, mitigate inflammation, and foster more favorable aging characteristics. Nonetheless, the empirical evidence concerning the direct impact of BCAAs on hippocampal plasticity and cognitive functioning is still insufficient and necessitates further exploration. The heterogeneity observed in intervention protocols, participant demographics, and outcome metrics emphasizes the intricacies inherent in the muscle–brain metabolic interface and underscores the imperative for more uniform methodologies. Subsequent inquiries ought to focus on larger, rigorously controlled clinical trials with extended intervention and follow-up durations to evaluate the persistence of these benefits. Research endeavors specifically oriented toward elucidating the mechanistic relationships linking BCAA supplementation, muscle metabolism, and neuroplasticity are of paramount importance, particularly with the incorporation of sensitive biomarkers indicative of neural function and cognitive processes. Furthermore, investigations should take into account the influence of individual variables such as genetic predispositions, baseline nutritional states, and coexisting health conditions in order to formulate personalized strategies for supplementation and exercise regimens. Examining the prospective synergistic interactions of BCAAs in conjunction with other nutrients or pharmacological agents may unveil novel pathways for alleviating age-related pathologies, including sarcopenia, cognitive deterioration, and neurodegenerative disorders. In summary, the amalgamation of interdisciplinary methodologies that integrate exercise science, nutrition, neurobiology, and geriatrics will be crucial to fully exploit the therapeutic efficacy of BCAAs in fostering healthy aging via the muscle–brain metabolic axis.
